# Canagliflozin alleviates high glucose-induced peritoneal fibrosis via HIF-1α inhibition

**DOI:** 10.3389/fphar.2023.1152611

**Published:** 2023-05-11

**Authors:** Jian Wang, Xin Lv, A-Shan-Jiang A-Ni-Wan, Sha-Sha Tian, Jun-Mei Wang, Hong-Yan Liu, Xiao-Guang Fan, Sai-Jun Zhou, Pei Yu

**Affiliations:** ^1^ NHC Key Laboratory of Hormones and Development, Chu Hsien-I Memorial Hospital and Tianjin Institute of Endocrinology, Tianjin Medical University, Tianjin, China; ^2^ Tianjin Key Laboratory of Metabolic Diseases, Tianjin Medical University, Tianjin, China; ^3^ Department of Nephrology, First Affiliated Hospital of Gannan Medical University, Ganzhou, China; ^4^ Department of Nephrology, Shanxi Bethune Hospital, Shanxi Academy of Medical Sciences, Tongji Shanxi Hospital, Third Hospital of Shanxi Medical University, Taiyuan, China; ^5^ Department of Nephrology, Henan Provincial People’s Hospital, Department of Nephrology of Central China Fuwai Hospital, Central China Fuwai Hospital of Zhengzhou University, Zhengzhou, China

**Keywords:** canagliflozin, hypoxia, peritoneal fibrosis, HIF-1α, TGF-β, Smad3

## Abstract

The cardioprotective effects of sodium-glucose cotransporter type 2 (SGLT2) inhibitors have been demonstrated in many studies. However, their benefits for end-stage kidney disease patients, particularly those on peritoneal dialysis, remain unclear. SGLT2 inhibition has shown peritoneal protective effects in some studies, but the mechanisms are still unknown. Herein, we investigated the peritoneal protective mechanisms of Canagliflozin *in vitro* by simulating hypoxia with CoCl_2_ in human peritoneal mesothelial cells (HPMCs) and rats by intraperitoneal injection of 4.25% peritoneal dialysate simulating chronic high glucose exposure. CoCl_2_ hypoxic intervention significantly increased HIF-1α abundance in HPMCs, activated TGF-β/p-Smad3 signaling, and promoted the production of fibrotic proteins (Fibronectin, COL1A2, and α-SMA). Meanwhile, Canagliflozin significantly improved the hypoxia of HPMCs, decreased HIF-1α abundance, inhibited TGF-β/p-Smad3 signaling, and decreased the expression of fibrotic proteins. Five-week intraperitoneal injection of 4.25% peritoneal dialysate remarkably increased peritoneal HIF-1α/TGF-β/p-Smad3 signaling and promoted peritoneal fibrosis and peritoneal thickening. At the same time, Canagliflozin significantly inhibited the HIF-1α/TGF-β/p-Smad3 signaling, prevented peritoneal fibrosis and peritoneal thickening, and improved peritoneal transportation and ultrafiltration. High glucose peritoneal dialysate increased the expression of peritoneal GLUT1, GLUT3 and SGLT2, all of which were inhibited by Canagliflozin. In conclusion, we showed that Canagliflozin could improve peritoneal fibrosis and function by ameliorating peritoneal hypoxia and inhibiting the HIF-1α/TGF-β/p-Smad3 signaling pathway, providing theoretical support for the clinical use of SGLT2 inhibitors in patients on peritoneal dialysis.

## 1 Introduction

Peritoneal dialysis (PD) is an important treatment approach for end-stage renal disease and has many benefits for clinical use compared to hemodialysis. These benefits include better preservation of residual kidney function, increased patient satisfaction, improved quality of life, delayed need for vascular access, enhanced anemia management, and reduced risk of blood-borne and respiratory viral infections, including COVID-19 (Bello et al., 2022). The current use of PD varies greatly worldwide, mostly applied in China, the United States, Mexico, and Thailand (Li et al., 2017; Bello et al., 2022). The important therapeutic goals of PD include protecting residual renal function, delaying the decline of peritoneal function, and reducing cardiovascular events in this high-risk population (Borkum et al., 2022). Sodium-glucose cotransporter type 2 (SGLT2) inhibitors are a class of drugs that lower blood glucose in diabetic patients by inhibiting renal tubular glucose uptake. Over the past few years, numerous studies have shown clear cardio-renal protective effects of SGLT2 inhibitors, independent of the glucose-lowering benefit. They can also reduce the risk of heart failure, delay the progression of renal failure, decrease the risk of acute kidney injury, reduce mean arterial pressure and potassium levels, and improve anemia (Mazidi et al., 2017; Mazer et al., 2020; Neuen et al., 2021; Ye et al., 2021; van der Aart-Van et al., 2022). However, whether these benefits can be extended to patients with more advanced CKD, particularly those on PD, is uncertain. Besides, the peritoneal protective effects of SGLT2 are controversial, according to available animal studies. Although SGLT2 was expressed in peritoneal mesothelial cells, Martus et al. showed that SGLT2 inhibitors failed to reduce peritoneal glucose uptake and increase ultrafiltration in an acute peritoneal dialysis rat model (Martus et al., 2021). In contrast, other chronic peritoneal dialysis animal models showed that SGLT2 inhibitors improved peritoneal fibrosis and function (Zhou et al., 2019; Balzer et al., 2020; Shentu et al., 2021). Therefore, novel studies are required to clarify the peritoneal protective mechanisms of SGLT2 inhibitors.

## 2 Materials and methods

### 2.1 Antibodies and chemicals

Antibodies against anti-HIF-1α (28b, sc-13515) and anti-ubiquitin (P4D1, sc-8017) were purchased from Santa Cruz Biotechnology; anti-Fibronectin (A12932), anti-COL1A2 (A5786), anti-α-SMA (A17910), and anti-β-Actin (AC038) from Abclonal (China); Anti-TGF-β (AF0297), Smad3 (AF1501), p-Smad3 (AF1759), and IgG (A7028) from Beyotime (China); anti-SGLT2 (24654-1-AP) from Proteintech (China). Horseradish peroxidase (HRP)-conjugated secondary goat anti-rabbit (AK1007) and goat anti-mouse (AK801X) antibodies, TRITC Goat anti-rabbit antibody (GR200G-39C), and FITC Goat anti-rabbit antibody (GR200G-02C) were purchased from SUNGENE BIOTECH (China). DAPI (S2110) and Canagliflozin (C5620) were from Solarbio (China), CoCl_2_ (7646-79-9) from Sigma-Aldrich, and MG-132 (T2154) from TargetMol.

### 2.2 Cell culture

We cultured human peritoneal mesothelial cells (HPMC cell line HMrSV5) in 1640 rpmi medium at 37°C in 5% CO_2_ supplemented with 10% fetal bovine serum and 1% penicillin/streptomycin. When HPMCs reached about 80% confluence, they were treated with a low-glucose medium (0.2% glucose, LG) or a high-glucose medium (2.5% glucose, HG). Pharmacological agents (Canagliflozin, CoCl_2,_ and MG-132) were supplemented in LG or HG medium at different concentrations as described in the figure legends. The CCK-8 kit (A1210, Solarbio, China) was used to detect cell viability, following the manufacturer’s instructions.

### 2.3 HIF-1α knockdown by siRNA transfection

At a cell confluence of 70%–90%, HPMCs were transfected with negative control (NC) siRNA (sense: UUC​UCC​GAA​CGU​GUC​ACG​UTT, antisense: ACG​UGA​CAC​GUU​CGG​AGA​ATT) or HIF-1α siRNA (sense: GCC​GCU​CAA​UUU​AUG​AAU​ATT, antisense: UAU​UCA​UAA​AUU​GAG​CGG​CTT), synthesized by GenePharma (China), using Lipofectamine 2000 transfection reagent (Invitrogen) according to the manufacturer’s instructions. After 24 h of transfection, the medium was replaced, and the cells were cultured for an additional 48 h in either a LG or HG medium or treated with 15 μM Canagliflozin.

### 2.4 Peritoneal fibrosis rat model and Canagliflozin administration

Male Sprague–Dawley rats (180–200 g, Spelford Biotechnology Co., Beijing, China) were housed in specific pathogen-free rooms at 24°C ± 2°C and humidity of 55% ± 5%. Rats had free access to water and a standard diet under continuous 12/12 h light/dark cycles. After 1 week of adaptive housing, we randomly divided rats into control (Con, n = 6), peritoneal dialysis (PD, n = 6), and Canagliflozin (Canag, n = 6) groups. Control and PD groups received daily intraperitoneal injections of 20 mL saline or 4.25% peritoneal dialysate through a syringe, respectively, for 5 weeks, while the Canagliflozin group was treated with an intraperitoneal injection of 10 mg/kg/d Canagliflozin based on the PD group. The dose of Canagliflozin refers to the report of Mishima et al. (Mishima et al., 2018). Animals were anesthetized with isoflurane at the end of the experiment and euthanized after performing peritoneal function tests and central venous blood collection for plasma creatinine testing. We collected the abdominal wall tissue and peritoneum outside the area of the intraperitoneal injection for subsequent testing.

### 2.5 Real-time quantitative PCR

Total RNA from HPMCs or rat peritoneum was extracted using a TRIzol Kit (10296028, ThermoFisher) following the manufacturer’s instructions. Total RNA was reverse-transcribed to cDNA using a qPCR Kit (R233, Vazyme, China). Real-time PCR reaction was performed using SYBR Green Fast qPCR Mix (RK21203, ABclonal, China) and analyzed by an applied biosystem from Thermo Fisher Scientific. Primers were synthesized by Sangon Biotech (Shanghai, China). The expression levels of target genes in each sample were normalized using β-Actin. The primers used for HPMCs were as follows: HIF-1α, F: ACT​GCA​CAG​GCC​ACA​TTC​ACG, R: AAT​CAG​CAC​CAA​GCA​GGT​CAT​AGG; SGLT2, F: GCT​GGA​ACA​TCT​ATG​CCT​CCG​T, R: TGA​CGA​AGG​TCT​GTA​CCG​TGT​C. β-Actin, F: GCT​CAC​CAT​GGA​TGA​TGA​TAT​CGC, R: CAC​ATA​GGA​ATC​CTT​CTG​ACC​CAT. The primers used for rat peritoneum were are listed below: GLUT1, F: TCC​ACC​ACA​CTC​ACC​ACA​CTC, R: GCC​TGC​CAA​AGC​GAT​TAA​CAA​AG; GLUT3, F: CAT​CTC​TGG​TGT​TCG​CTG​TTA​CTG, R: GTC​TTC​CAA​CCG​CTC​TTC​CAA​C; SGLT2, F: CAG​CAG​CAG​CAC​ACT​CTT​CAC, R: AGC​CAA​GCC​ACG​GAC​ACT​G; β-Actin, F: CTA​TCG​GCA​ATG​AGC​GGT​TCC; R: GCA​CTG​TGT​TGG​CAT​AGA​GGT​C.

### 2.6 Immunoblot analysis

Proteins were extracted from cultured cells or rat peritoneum using RIPA buffer (R0010, Solarbio, China) with protease and phosphatase inhibitors. Lysates were collected after centrifugation at 14,000 *g* and 4 C for 5 min, and protein concentrations were determined using the BCA Protein Assay Kit (PC0020, Solarbio, China). Then, supernatants were heated at 100°C for 10 min with an SDS-PAGE loading buffer. Equal protein amounts were separated by SDS–polyacrylamide gel electrophoresis and electrotransferred to nitrocellulose membranes (Merck Millipore Ltd.). Membranes were blocked with 5% nonfat milk and incubated overnight with primary antibodies at 4°C, followed by 1-h incubation with secondary antibodies at room temperature. Proteins were visualized with an ECL chemiluminescent substrate by GelView 6,000 Pro system (BLT, China).

### 2.7 Immunoprecipitation

Cells were lysed on ice with RIPA buffer (R0010, Solarbio, China) with protease and phosphatase inhibitors. Lysates were harvested after centrifugation at 14,000 *g* and 4°C for 5 min. Then, primary antibodies against HIF-1α and IgG were incubated with protein A/G magnetic beads (HY-K0202, MCE) for 30 min on a rotary shaker. Beads were washed with 0.2% PBST and incubated with the supernatant on a rotary shaker for 30 min. Beads were washed again and eluted with SDS-PAGE loading buffer at 100°C for 10 min. Finally, the supernatant was collected for SDS-PAGE.

### 2.8 Immunofluorescence

Cells or frozen sections of rat abdominal wall tissues were fixed with 4% paraformaldehyde for 15 min and permeabilized in 0.5% Triton X-100/PBS for 15 min at room temperature. Then, they were blocked with 5% bovine serum albumin/PBS and incubated with a primary antibody overnight at 4°C. On the next day, samples were incubated with a fluorescent secondary antibody at 37°C for 1 h, followed by nuclei DAPI staining with anti-fluorescence quenching sealing liquid (S2110, Solarbio, China). Samples were visualized under an OLYMPUS IX83 fluorescence microscope (Olympus Co., Japan).

### 2.9 Peritoneal function test

After 5 weeks of intraperitoneal injection, rats received a two-day rest period to absorb the injected fluid better. Then, rats were intraperitoneally injected with 20 mL of 4.25% peritoneal dialysate. After 2 h, rats were anesthetized with isoflurane, the abdominal cavity was opened along the midline, and the peritoneal fluid was emptied and collected. The ultrafiltration volume was calculated as the fluid drained from the abdominal cavity minus 20 mL. Finally, glucose, sodium and creatinine concentrations in the peritoneal outflow were measured.

### 2.10 Masson trichrome staining

First, abdominal wall samples were fixed in 4% paraformaldehyde, and paraffin-embedded sections were prepared. Sections were stained using a Masson trichrome staining kit (G1340, Solarbio, China) following the manufacturer’s protocol. Photographs were taken using an OLYMPUS IX83 fluorescence microscope (Olympus Co., Japan), and peritoneal thickness was measured using OLYMPUS cellSens Standard 2.1 (Olympus Co., Japan).

### 2.11 Immunohistochemistry staining

Briefly, paraffin-embedded sample sections were dewaxed, hydrated, and treated with 3% hydrogen peroxide at room temperature for 10 min. Subsequently, sections were heat-treated with Tris-EDTA buffer to repair the antigen and blocked with 5% bovine serum albumin/PBS for 30 min at room temperature, followed by overnight incubation with primary antibodies at 4°C. The next day, sections were washed with PBS and incubated with HRP-conjugated secondary antibodies for 1 h at 37°C. Then, sections were stained with DAB for 1–10 min and restained with hematoxylin. An OLYMPUS IX83 fluorescence microscope (Olympus Co., Japan) was used to view and photograph samples.

### 2.12 Statistical analysis

Data are shown as means ± SEM and were analyzed using GraphPad Prism 8. Two or more groups were compared and analyzed by analysis of variance (ANOVA) followed by Tukey’s multiple comparisons tests. A *p* < 0.05 was considered statistically significant.

## 3 Results

### 3.1 SGLT2 expression in HPMCs and pharmaceutical effects on cell viability

**FIGURE 1:**
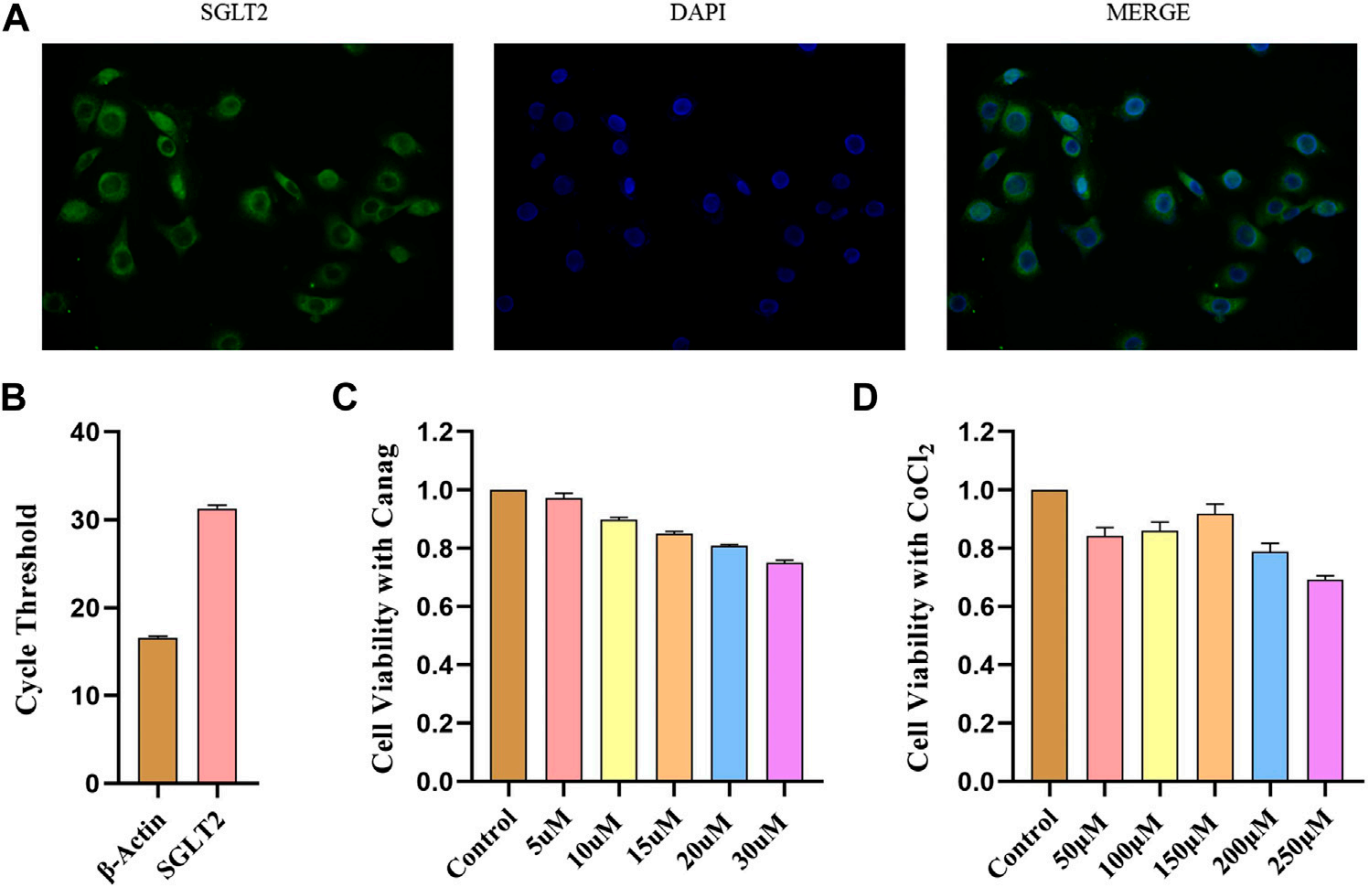
SGLT2 expression in HPMCs and CCK-8 viability test. **(A)** Immunofluorescence detection of SGLT2 expression in HPMCs. **(B)** SGLT2 mRNA expression in HPMCs detected by real-time quantitative PCR. **(C)** CCK-8 assay to test the effects of different Canagliflozin concentrations on HPMCs viability. **(D)** HPMCs viability under different CoCl_2_ concentrations by CCK-8 test. Canag: Canagliflozin.

**FIGURE 2:**
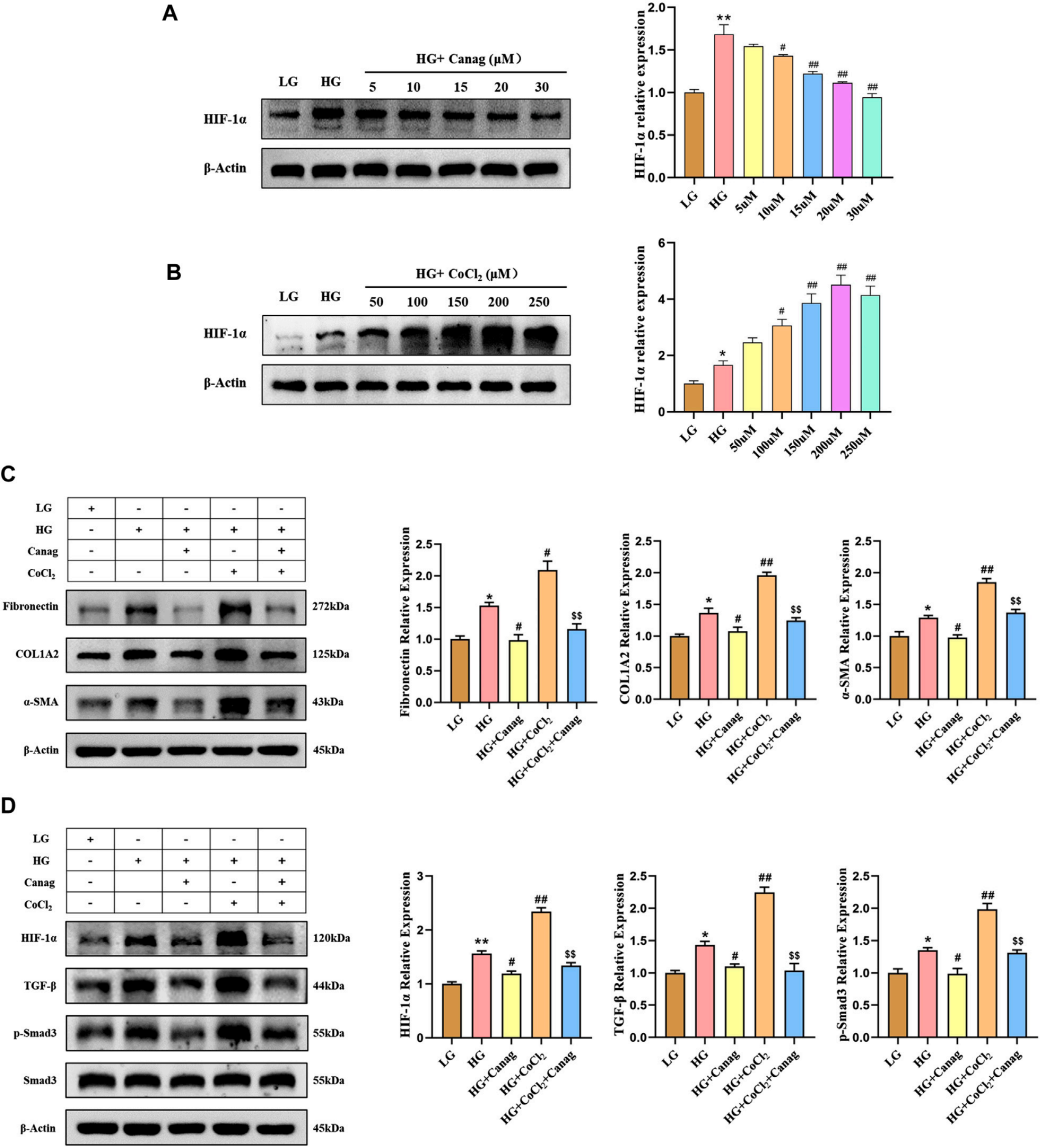
Canagliflozin inhibits HPMCs fibrosis and TGF-β/p-Smad3 signaling induced by high glucose and HIF-1α. **(A, B)** Immunoblotting and HIF-1α abundance quantification at different concentrations of Canagliflozin or CoCl_2_ for 48 h. **(C)** Immunoblotting and quantification of fibrotic proteins at 15 μΜ Canagliflozin under high glucose with or without 150 μΜ CoCl_2_ for 48 h. **(D)** Immunoblotting and quantification of HIF-1α, TGF-β, Smad3, and p-Smad3 influenced by 15 μΜ Canagliflozin under high glucose with or without 150 μΜ CoCl_2_ for 48 h *, *p* < 0.05; **, *p* < 0.001 vs LG group. ^#^, *p* < 0.05; ^##^, *p* < 0.001 vs HG group. ^$^, *p* < 0.05; ^$$^, *p* < 0.001 vs HG + CoCl_2_ group. LG: 0.2% glucose medium; HG: 2.5% glucose medium; Canag: Canagliflozin.

The immunofluorescence revealed the presence of SGLT2 in the membranes of HPMCs (Figure 1A). Real-time quantitative PCR also validated the expression of SGLT2 in HPMCs. However, the expression level was relatively low due to high cycle threshold values, as shown in Figure 1B. We used different Canagliflozin concentrations to find a suitable intervention dose and performed cell viability tests. The CCK-8 assay showed that Canagliflozin dose-dependently reduced the viability of HPMCs (Figure 1C). The cell viability was greater than 80% at 15 μΜ and it has a well inhibitory effect on HIF-1α (Figure 2A), this dose was chosen for subsequent cellular experiments. CoCl_2_ was used to simulate hypoxia and raise HIF-1α. We found that 150 μΜ CoCl_2_ had a much smaller effect on cell viability (Figure 1D) and it significantly increased HIF-1α abundance (Figure 2B), thereby used as the subsequent intervention condition.

### 3.2 Canagliflozin inhibits HPMCs fibrosis and TGF-β/p-Smad3 signaling induced by high glucose and HIF-1α

Hypoxia and elevated HIF-1α are important contributors to peritoneal fibrosis during PD and Canagliflozin was reported to have an inhibitory effect on HIF-1α in diabetic nephropathy. Herein, we cultured HPMCs with different concentrations of Canagliflozin or CoCl_2_ to observe their effects on HIF-1α. Canagliflozin evidently reduced the abundance of HIF-1α in a dose-dependent manner (Figure 2A), while CoCl_2_ increased HIF-1α abundance in a dose-dependent trend at concentrations less than 250 μΜ (Figure 2B). Combining the results of CCK-8 viability test and Western Blot experiments, 15 μΜ Canagliflozin and 150 μΜ CoCl_2_ were chosen for subsequent experiments. The following immunoblotting results showed that high glucose increased the expression of fibrotic proteins (Fibronectin, COL1A2, and α-SMA) and the abundance of HIF-1α, TGF-β, and p-Smad3, all inhibited by Canagliflozin (Figures 2C,D). Furthermore, CoCl_2_ increased the abundance of HIF-1α on the basis of high glucose and promoted the expression of TGF-β, p-Smad3 and fibrotic proteins. Canagliflozin significantly reduced CoCl_2_-induced HIF-1α abundance, inhibited TGF-β/p-Smad3 signaling, and suppressed HPMC fibrosis (Figures 2C,D). To provide additional evidence that Canagliflozin acts through HIF-1α, we conducted HIF-1α knockdown experiments in HPMCs. As depicted in Supplementary Figure S1, HIF-1α knockdown suppressed the upregulation of fibrosis-related proteins (Fibronectin, COL1A2, α-SMA, TGF-β, and p-Smad3) induced by high glucose. The co-treatment of Canagliflozin with HIF-1α knockdown did not further inhibit the expression of fibrosis-related proteins, indicating that Canagliflozin exerts its effects via the HIF-1α pathway.

### 3.3 Canagliflozin promotes HIF-1α ubiquitination and proteasomal degradation in HPMCs

**FIGURE 3:**
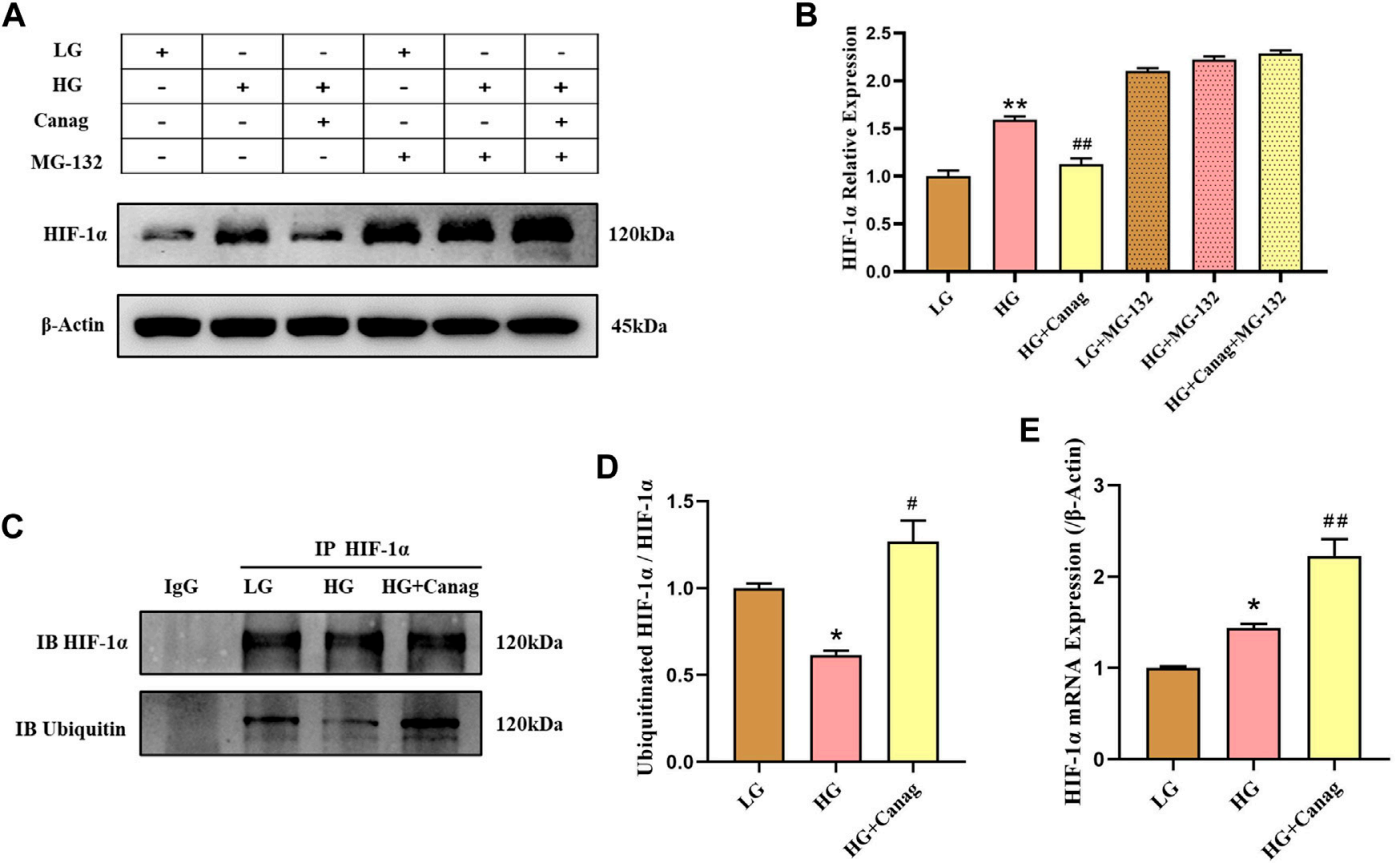
Canagliflozin promotes HIF-1α ubiquitination and proteasomal degradation in HPMCs. **(A, B)** Immunoblotting and quantification of HIF-1α under high glucose with or without 15 μΜ Canagliflozin for 48 h. MG-132 (0.1 μΜ) was added in the last 24 h **(C, D)** Immunoprecipitated HIF-1α, immunoblots, and quantification of total and ubiquitinated HIF-1α. **(E)** HIF-1α mRNA levels under LG and HG or with 15 μΜ Canagliflozin for 48 h *, *p* < 0.05; **, *p* < 0.001 vs LG group. ^#^, *p* < 0.05; ^##^, *p* < 0.001 vs HG group. LG: 0.2% glucose medium; HG: 2.5% glucose medium; Canag: Canagliflozin.

Canagliflozin inhibited HIF-1α abundance induced by high glucose and CoCl_2_ but could not decrease the HIF-1α mRNA level (Figure 3E). Hence, we hypothesized that Canagliflozin might promote HIF-1α degradation. The addition of the proteasome inhibitor MG-132 significantly increased HIF-1α levels in low glucose and Canagliflozin groups (Figures 3A,B), indicating that HIF-1α proteasomal degradation was higher in the two groups compared to the high glucose group, and Canagliflozin promoted the proteasomal degradation of HIF-1α. Immunoprecipitation and immunoblotting showed higher ubiquitination levels of HIF-1α in the low glucose and Canagliflozin groups compared to the high glucose group (Figures 3C,D), suggesting that Canagliflozin increased HIF-1α ubiquitination. These results indicated that Canagliflozin increased ubiquitination HIF-1α levels and promoted its proteasomal degradation.

### 3.4 Canagliflozin prevents peritoneal thickness and improves peritoneal function in rats

**FIGURE 4:**
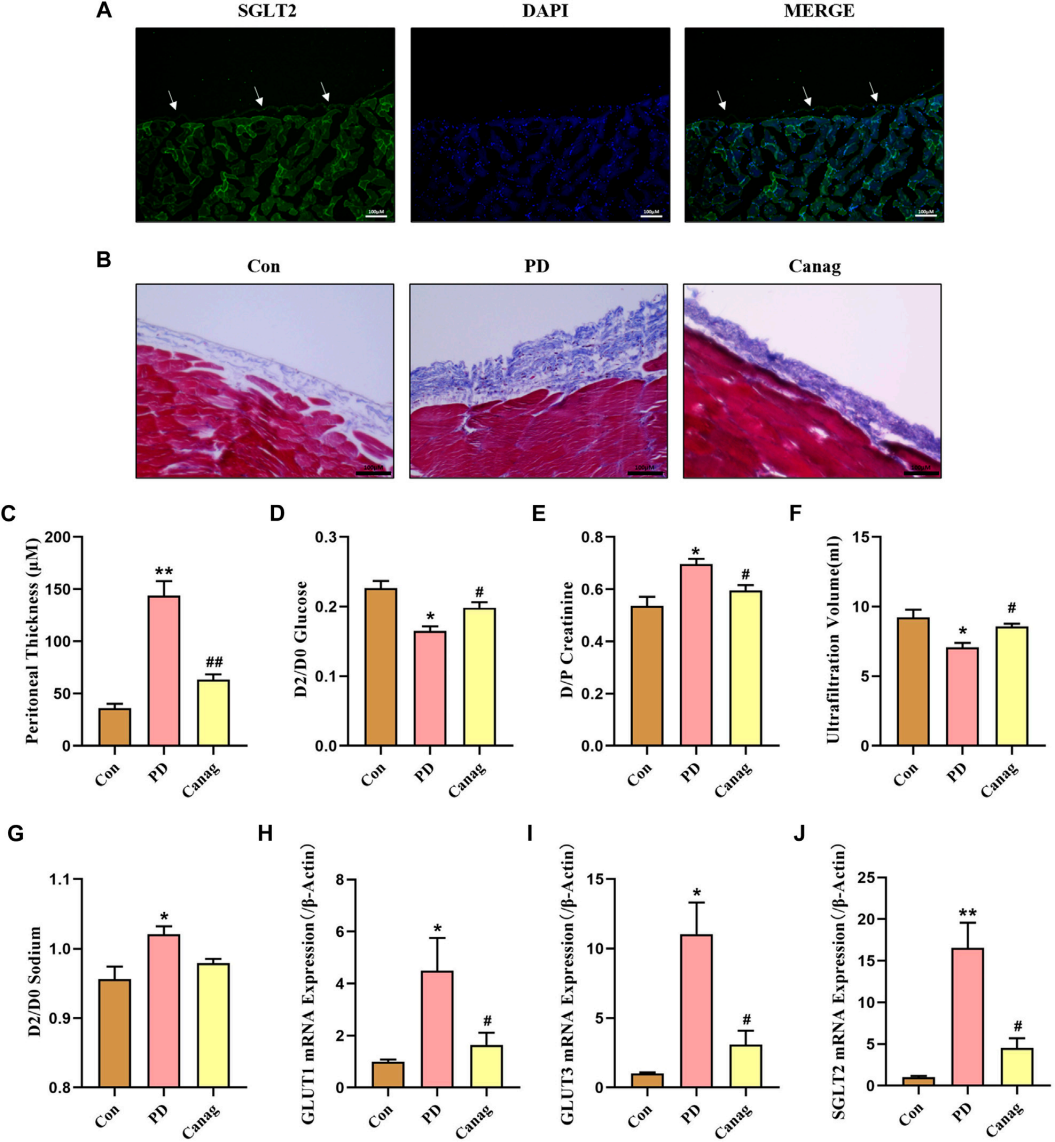
Canagliflozin prevents peritoneal -thickness and improves peritoneal function in rats. **(A)** Immunofluorescence detection of SGLT2 expression in the peritoneal membrane. **(B)** Masson trichrome staining of rat peritoneal membrane in different groups. **(C)** Thickness quantitative analysis of the rat peritoneum in each group. **(D–G)** Peritoneal function based on glucose absorption (glucose concentration ratio of 2 and 0-h peritoneal dialysate, D2/D0), creatinine transportation (creatinine concentration ratio of 2-h peritoneal dialysate and plasma, D/P), ultrafiltration and sodium transportation (sodium concentration ratio of 2 and 0-h peritoneal dialysate, D2/D0) in each group of rats. **(H–J)** Peritoneal mRNA expressions of GLUT1, GLUT3 and SGLT2 detected by real-time quantitative PCR. *, *p* < 0.05; **, *p* < 0.001 vs Con group. ^#^, *p* < 0.05; ^##^, *p* < 0.001 vs PD group.

Furthermore, we evaluated SGLT2 expression in the rat’s abdominal wall. The immunofluorescence showed that SGLT2 was expressed in the peritoneal membrane and skeletal muscle (Figure 4A). Then, rats received daily intraperitoneal injections of peritoneal dialysate to simulate PD. The Masson trichrome staining and quantitative analysis showed that the peritoneal membrane of PD rats was significantly thickened, and collagen fibers were greatly increased compared to the saline control group. Canagliflozin treatment inhibited the peritoneal thickening and collagen fiber deposit induced by peritoneal dialysate (Figures 4B,C). Peritoneal function tests showed that Canagliflozin inhibited glucose uptake and creatinine hypertransportation and increased peritoneal ultrafiltration compared to the PD group (Figures 4D–F). The sodium concentration in the dialysate increased among the PD group. However, treatment with Canagliflozin resulted in a decrease in sodium concentration, compared with PD group, although this reduction was not statistically significant (Figure 4G). The peritoneal mRNA expressions of GLUT1, GLUT3 and SGLT2 in the PD group exhibited a significant increase, with particularly high levels observed for GLUT3 and SGLT2. Treatment with Canagliflozin was found to effectively suppress the increase in both GLUTs and SGLT2 mRNA levels (Figures 4H–J)

### 3.5 Canagliflozin inhibits fibrotic proteins expression in the peritoneal membrane of rats

**FIGURE 5:**
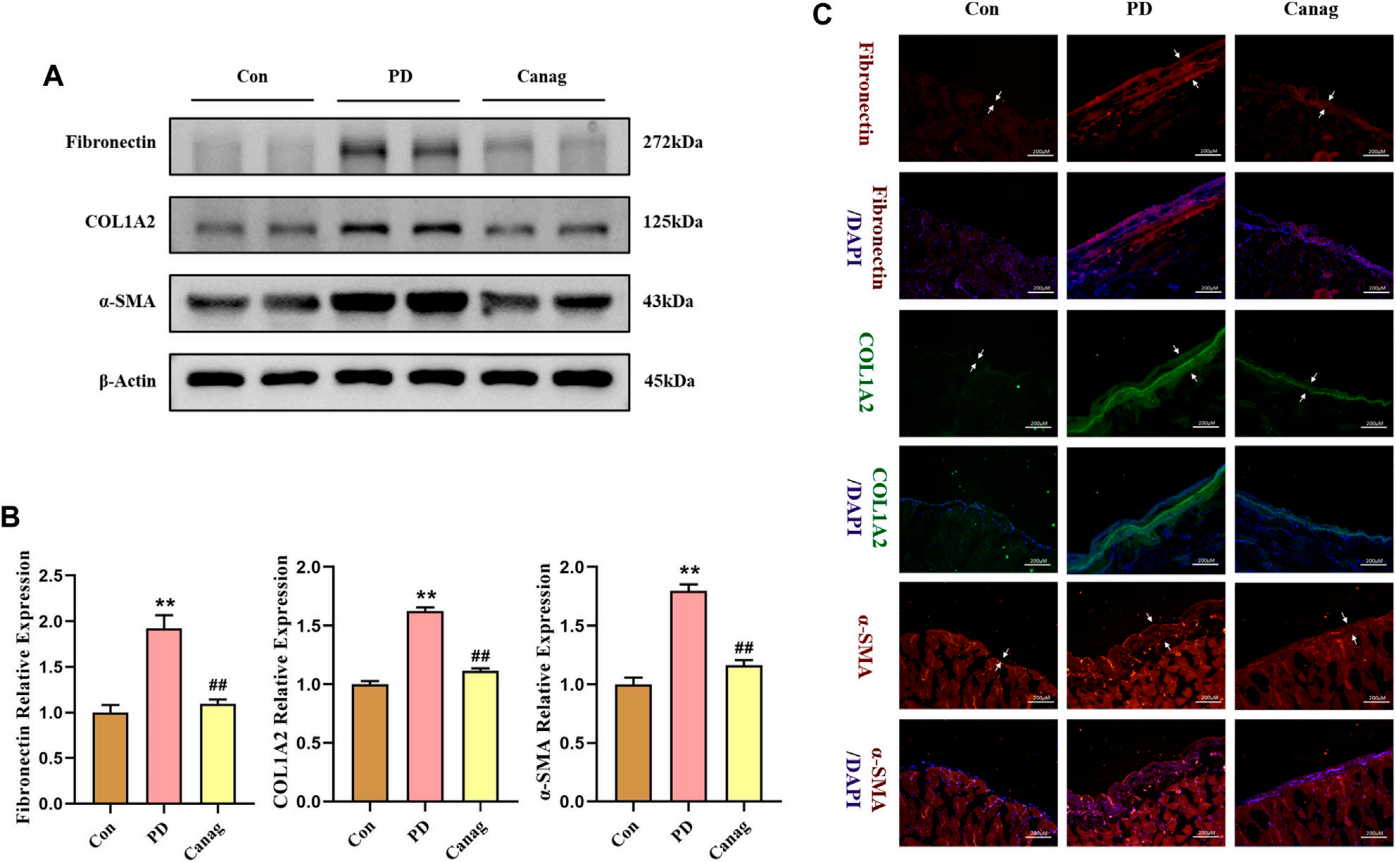
Canagliflozin inhibits the fibrotic protein expression in the peritoneal membrane of rats. **(A, B)** Immunoblotting and quantification of fibrotic proteins in peritoneal membrane. The representative image shows two out of six rat peritoneal protein samples from each group, which were all detected and included in the statistical analysis. **(C)** Fibrotic proteins deposit in the peritoneal membrane detected immunofluorescence. **, *p* < 0.001 vs Con group; ^#^^#^, *p* < 0.001 vs PD group.

Moreover, the immunoblotting and quantitative analysis revealed that peritoneal dialysate increased the expression of peritoneal fibrosis proteins (Fibronectin, COL1A2, and α-SMA) in rats, while Canagliflozin treatment decreased their levels (Figures 5A,B). The immunofluorescence showed a significant increase in the deposition of Fibronectin, COL1A2, and α-SMA in the peritoneal membrane, which was decreased by Canagliflozin (Figure 5C).

### 3.6 Canagliflozin decreases the HIF-1α abundance and inhibits TGF-β/p-Smad3 signaling of peritoneal membrane in rats

**FIGURE 6:**
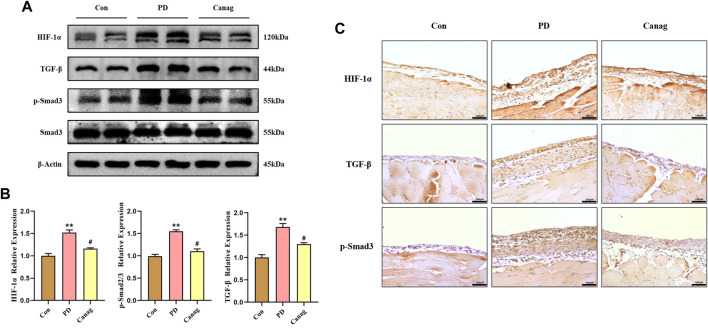
Canagliflozin decreases HIF-1α abundance and inhibits TGF-β/p-Smad3 signaling in the peritoneal membrane of rats. **(A, B)** Immunoblotting and HIF-1α, TGF-β, p-Smad3, and Smad3 quantification in the peritoneal membrane. The representative image shows two out of six rat peritoneal protein samples from each group, which were all detected and included in the statistical analysis. **(C)** Immunohistochemistry of HIF-1α, TGF-β, and p-Smad3 in the peritoneal membrane. **, *p* < 0.001 vs Con group. ^#^, *p* < 0.05 vs PD group.

TGF-β/Smad is a downstream signaling pathway of HIF-1α and are important molecules promoting fibrosis. The immunoblotting and quantitative analysis of peritoneal proteins showed that peritoneal dialysate increased HIF-1α abundance while promoting TGF-β and p-Smad3 levels. Consistent with cellular experiments, Canagliflozin significantly decreased the abundance of HIF-1α and inhibited TGF-β and p-Smad3 (Figures 6A,B). Additionally, the peritoneal membrane immunohistochemical examination also displayed an increased abundance of HIF-1α, TGF-β, and p-Smad3 in the PD group, which were inhibited by Canagliflozin (Figure 6C).

## 4 Discussion

Peritoneal dialysis (PD) is one of the renal replacement therapies for end-stage renal disease patients and has the advantages of home dialysis and cost savings compared to hemodialysis. The convenience of home dialysis is particularly evident during the COVID-19 epidemic. However, chronic high glucose exposure of the peritoneum is not physiological, leading to peritoneal fibrosis and dysfunction, which is a major factor in PD failure, leading to the transfer of patients to hemodialysis or death (Balzer, 2020). Krediet recently proposed the glucose/hypoxia/GLUT-1 hypothesis to explain why prolonged exposure to high glucose could lead to peritoneal fibrosis and decreased ultrafiltration (Krediet, 2021; Krediet and Parikova, 2022). Glucose absorption causes an increase in the intracellular NADH/NAD^+^ ratio, called pseudohypoxia. Intracellular hypoxia upregulates the hypoxia-inducible factor-1 gene, which stimulates the glucose transporter-1 (GLUT-1) gene and many profibrotic genes (e.g., TGF-β, VEGF, PAI-1, and CTGF), promoting the development of peritoneal fibrosis. Increased GLUT-1 expression of myofibroblasts leads to a decreased crystalloid osmotic pressure gradient for the endothelial water channel aquaporin-1, finally resulting in decreased ultrafiltration.

Moreover, HIF-1α is the active subunit of HIF-1, a key factor in response to hypoxic stress (Ke and Costa, 2006). The participation of hypoxia and HIF-1α in the development of peritoneal fibrosis has been previously demonstrated. Manuprasert et al. reported that intermittent hypoxia could induce HIF-1α abundance and enhance peritoneal membrane thickness in rats (Manuprasert et al., 2022). Hypoxia also increases the abundance of HIF-1α, Snail-1, VEGF, and MMP-2 in HPMCs, while increasing mesenchymal cell markers and decreasing epithelial cell markers (Morishita et al., 2016). Additionally, blocking the hypoxic response or HIF-1α inhibition could mitigate peritoneal fibrosis (Sekiguchi et al., 2012; Wang et al., 2020).

SGLT2 inhibitors are a class of drugs that decrease blood glucose by inhibiting renal tubular glucose uptake and increasing urinary glucose excretion. Their cardio-renal protective effects have been widely demonstrated (van der Aart-Van et al., 2022). The current mainstream peritoneal dialysate still uses high glucose as the osmotic medium. As the time of PD increases, peritoneal angiogenesis and thickening progress, glucose absorption increases, and the glucose osmotic gradient decreases, resulting in less ultrafiltration. Given the role of SGLT2 inhibitors in reducing renal tubular glucose uptake, many physicians in the field of PD are actively exploring whether SGLT2 is expressed in the peritoneum and whether SGLT2 inhibitors can inhibit peritoneal glucose uptake to maintain osmolality and increase ultrafiltration. SGLT2 is expressed in humans, rats, and mice peritoneum, and SGLT2 inhibitors can suppress glucose uptake and increase ultrafiltration (Zhou et al., 2019; Balzer et al., 2020; Shentu et al., 2021). However, Martus et al. showed that SGLT2 inhibitors did not inhibit peritoneal glucose uptake and increase ultrafiltration (Martus et al., 2021). Therefore, the peritoneal protective effect of SGLT2 inhibitors remains controversial. It is generally accepted that glucose uptake during PD occurs mainly via cellular bypass transport. Although SGLT2 expression is present in peritoneal mesothelial cells, the amount of glucose transported should be minimal and have little effect on the overall amount of glucose in the peritoneal dialysate. This explanation is consistent with the findings of Martus et al. that SGLT2 inhibitors do not noticeably inhibit glucose uptake and increase ultrafiltration (Martus et al., 2021). However, SGLT2 inhibitors improved peritoneal fibrosis and function in animal models of chronic high glucose exposure (Zhou et al., 2019; Balzer et al., 2020; Shentu et al., 2021). Thus, SGLT2 inhibitors might improve peritoneal function indirectly by improving peritoneal fibrosis. Since high glucose-induced peritoneal pseudohypoxia and elevated HIF-1α are important mechanisms of peritoneal fibrosis and dysfunction, and SGLT2 inhibitors can improve renal fibrosis by ameliorating renal hypoxia and inhibiting HIF-1α in diabetic nephropathy (Bessho et al., 2019; Packer, 2021; Inada et al., 2022), we hypothesized that SGLT2 inhibitors might improve peritoneal function by ameliorating peritoneal hypoxia, decreasing HIF-1α levels, and inhibiting high glucose-induced peritoneal fibrosis.

Driven by this hypothesis, we used CoCl_2_ to simulate hypoxia in peritoneal mesothelial cell culture to explore the peritoneal protective mechanism of Canagliflozin. The abundance of HIF-1α and fibrotic proteins significantly increased by high glucose, and CoCl_2_ further elevated HIF-1α and promoted fibrosis of peritoneal mesothelial cells. Canagliflozin inhibited the increase of HIF-1α induced by high glucose and CoCl_2_ and prevented HPMCs fibrosis. Knockdown of HIF-1α abolishes the inhibitory effect of Canagliflozin on fibrosis. These results suggested that the main mechanism of Canagliflozin against peritoneal fibrosis was the improvement of hypoxia and HIF-1α inhibition. TGF-β/Smad3 signaling plays an important role during peritoneal fibrosis (Patel et al., 2010; Shentu et al., 2021). Herein, we examined TGF-β/Smad3 protein levels to investigate whether they participated in CoCl_2_ hypoxia-induced peritoneal fibrosis and the therapeutic mechanisms of Canagliflozin. TGF-β and p-Smad3 levels increased under high glucose and CoCl_2_ hypoxic conditions, inhibited by Canagliflozin. These results suggested that Canagliflozin also ameliorates peritoneal fibrosis by suppressing HIF-1α and the downstream TGF-β/p-Smad3 signaling. Then, we tested the peritoneal protective effect of Canagliflozin in rats. Five weeks of chronic high glucose exposure significantly increased peritoneal thickness and increased the abundance of HIF-1α, TGF-β, p-Smad3, and fibrotic proteins. Consistent with the cellular experiments, Canagliflozin inhibited the HIF-1α/TGF-β/p-Smad3 signal pathway, reduced peritoneal deposition of fibrotic proteins, and prevented peritoneal thickening. Canagliflozin also improved peritoneal function, reduced glucose uptake, decreased creatinine transportation, and increased ultrafiltration.

Studies have demonstrated expression of GLUT1, GLUT3 and SGLT2 in peritoneal mesothelial cells or peritoneal membrane (Fischereder et al., 2003; Debray-Garcia et al., 2016; Zhou et al., 2019; Balzer et al., 2020; Schricker et al., 2022), although the results have been inconsistent across studies. These transporters were predominantly located adjacent to the vessel walls of the peritoneal membrane (Schricker et al., 2022). Our results showed that high-glucose peritoneal dialysate increased the mRNA levels of rat peritoneal GLUT1, GLUT3, and SGLT2, with the mRNA levels of GLUT3 and SGLT2 being significantly increased in particular. All the mRNA levels were decreased by Canagliflozin treatment. We interpret this result as an increase in HIF-1α promoting peritoneal angiogenesis and fibrosis, increasing the expression of GLUTs and SGLT2. Canagliflozin inhibits the production of HIF-1α, leading to a reduction in peritoneal angiogenesis and fibrosis, while also decreasing the expression of GLUTs and SGLT2, ultimately resulting in a decrease in glucose uptake. Although Canagliflozin reduces the expression of SGLT2, its mechanism for inhibiting peritoneal glucose absorption may not be directly through SGLT2, as it did not increase the sodium level in peritoneal dialysate. The effects of SGLT2 inhibitors on peritoneal GLUTs and SGLTS have been inconsistently reported. Balzer et al. found that Dapagliflozin inhibited peritoneal SGLT2 upregulation in PD mice but had no effect on elevated GLUT1 and GLUT3 (Balzer et al., 2020). Experiments by Shentu et al. showed that Empagliflozin inhibited the increase of rat peritoneal SGLT2 expression induced by PD (Shentu et al., 2021). However, Zhou et al. reported an increase in peritoneal SGLT2 expression induced by Empagliflozin (Zhou et al., 2019). The inconsistencies in the different experimental results may be related to the varying degrees of peritoneal fibrosis and transport status induced by diverse interventions. Additionally, different SGLT2 inhibitors may have pharmacological differences due to variations in peritoneal local drug concentration and duration of action.

Previous animal studies have indicated that SGLT2 inhibitors can improve peritoneal fibrosis and function caused by chronic high glucose exposure. However, their efficacy and safety in clinical patients under PD need further exploration. Notably, SGLT2 inhibitors have been used in diabetic patients under PD and achieved good clinical outcomes. Six months of dapagliflozin increased peritoneal ultrafiltration and urine output and improved inflammatory parameters with no significant effect on peritoneal Kt/V or residual renal function (K Alhwiesh et al., 2022). Additionally, trials have shown multiple potential cardiorenal benefits of SGLT2 inhibitors. However, these studies have excluded patients with glomerular filtration rates below 25 mL/min/1.73 m^2^ (van der Aart-Van et al., 2022). Dialysis patients are at the highest risk of cardiovascular disease and would benefit most from effective cardioprotective therapy, an important reason to actively explore the use of SGLT2 inhibitors in PD (Borkum et al., 2022). Existing animal and clinical trials are insufficient to confirm and expand the clinical use of SGLT2 inhibitors in PD patients. Therefore, more clinical studies are required to explore better their clinical benefits and safety.

## 5 Conclusion

In summary, we demonstrated that Canagliflozin prevents peritoneal fibrosis induced by high glucose and improves peritoneal function by alleviating peritoneal hypoxia and inhibiting HIF-1α and TGF-β/p-Smad3 signaling pathways.

## Data Availability

The original contributions presented in the study are included in the article/Supplementary Material, further inquiries can be directed to the corresponding author.
