# Supplementary material for: Canagliflozin alleviates high glucose-induced peritoneal fibrosis via HIF-1α inhibition
Source: Front Pharmacol. 2023 May 11;14:1152611. doi: 10.3389/fphar.2023.1152611 (PMC10213900; doi:10.3389/fphar.2023.1152611)
Supplement: Supplementary file 1 [file DataSheet1.docx]

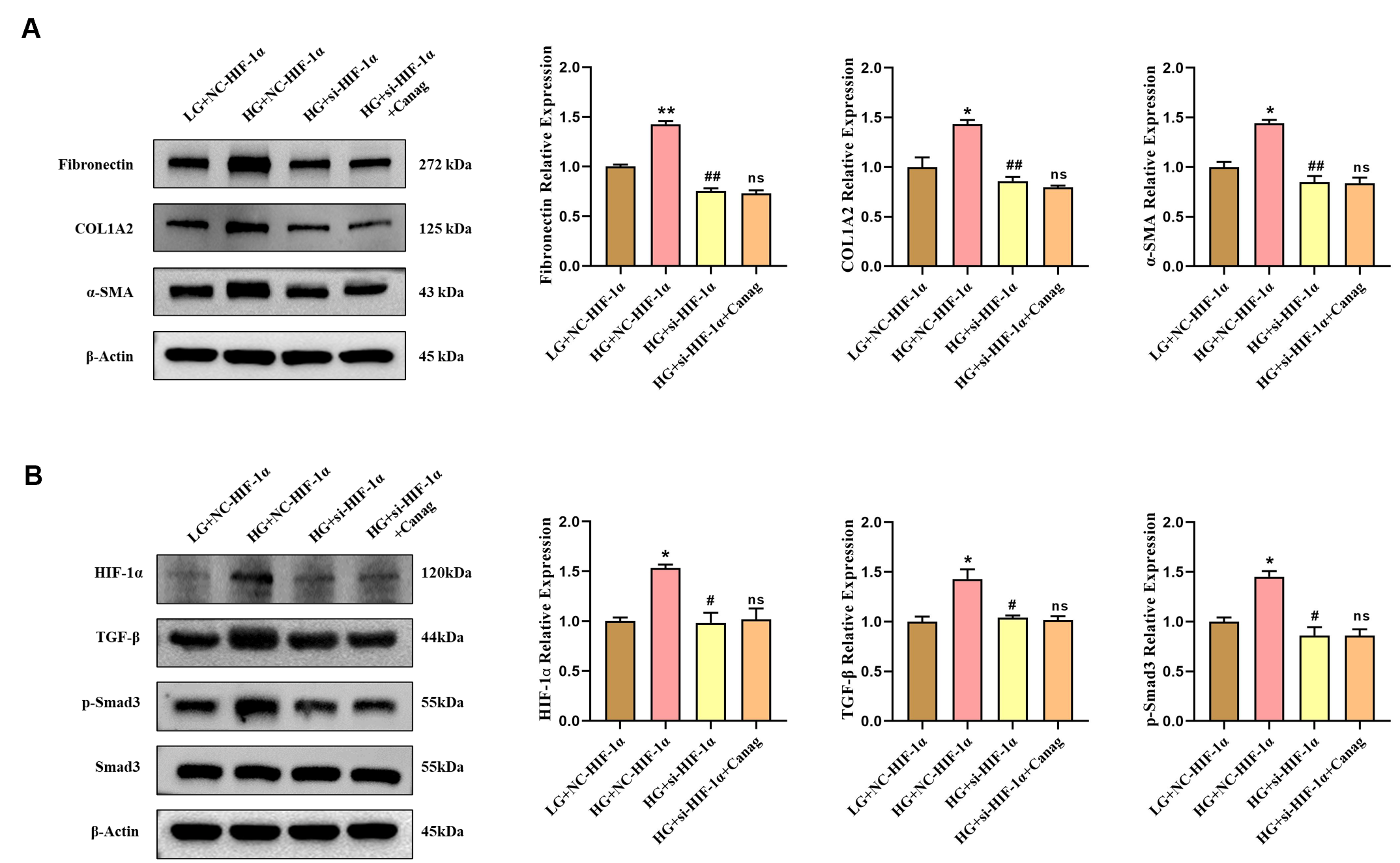


Supplementary Figure S1. Knockdown of HIF-1α abolishes the inhibitory effect of Canagliflozin on fibrosis. (A) Immunoblotting and quantification of fibrotic proteins under conditions of HIF-1α knockdown with or without 15 μΜ Canagliflozin for 48 h. (B) Immunoblotting and quantification of HIF-1α, TGF-β, Smad3, and p-Smad3 under conditions of HIF-1α knockdown with or without 15 μΜ Canagliflozin for 48 h. *, *p* < 0.05; **, *p* < 0.001 vs. LG+NC-HIF-1α group. ^#^, *p* < 0.05; ^##^, *p* < 0.001 vs. HG+NC-HIF-1α group. ^ns^, *p* > 0.05 vs. HG+si-HIF-1α group, no statistical difference. LG: 0.2% glucose medium; HG: 2.5% glucose medium; Canag: Canagliflozin.
